# Evaluation of molecular inversion probe versus TruSeq® custom methods for targeted next-generation sequencing

**DOI:** 10.1371/journal.pone.0238467

**Published:** 2020-09-02

**Authors:** Rowida Almomani, Margherita Marchi, Maurice Sopacua, Patrick Lindsey, Erika Salvi, Bart de Koning, Silvia Santoro, Stefania Magri, Hubert J. M. Smeets, Filippo Martinelli Boneschi, Rayaz R. Malik, Dan Ziegler, Janneke G. J. Hoeijmakers, Gidon Bönhof, Sulayman Dib-Hajj, Stephen G. Waxman, Ingemar S. J. Merkies, Giuseppe Lauria, Catharina G. Faber, Monique M. Gerrits, on behalf on the PROPANE Study Group

**Affiliations:** 1 Department of Genetics and Cell Biology, Clinical Genomics Unit, Maastricht University, Maastricht, The Netherlands; 2 MHeNs school of Mental Health and Neuroscience, Maastricht University, Maastricht, The Netherlands; 3 Department of Medical Laboratory Sciences, Jordan University of Science and Technology, Irbid, Jordan; 4 Neuroalgology Units, Fondazione IRCCS Istituto Neurologico “Carlo Besta” Milan, Milan, Italy; 5 Department of Neurology, Maastricht University Medical Center+, Maastricht, the Netherlands; 6 Department of Clinical Genetics, Maastricht University Medical Center+, Maastricht, the Netherlands; 7 Laboratory of Human Genetics of Neurological Disorders, Institute of Experimental Neurology (INSPE), Division of Neuroscience, San Raffaele Scientific Institute, Milan, Italy; 8 Institute of Human Development, Centre for Endocrinology and Diabetes, University of Manchester and Central Manchester NHS Foundation Trust, Manchester Academic Health Science Center, Manchester, United Kingdom; 9 Department of Medicine, Weill Cornell Medicine, Doha, Qatar; 10 Institute for Clinical Diabetology, German Diabetes Center, Leibniz Center for Diabetes Research, Düsseldorf, Germany; 11 Department of Endocrinology and Diabetology, Medical Faculty, Heinrich Heine University, Düsseldorf, Germany; 12 Department of Neurology, Yale University School of Medicine, Yale, New Haven, United States of America; 13 Center for Neuroscience and Regeneration Research, Yale University School of Medicine, Yale, New Haven, United States of America; 14 Center for Neuroscience and Regeneration Research, Veterans Affairs Medical Center, West Haven, Connecticut, United States of America; 15 Department of Neurology, St Elisabeth Hospital, Willemstad, Curaçao; 16 Department of Biomedical and Clinical Sciences "Luigi Sacco", University of Milan, Milan, Italy; University of Hong Kong, HONG KONG

## Abstract

Resolving the genetic architecture of painful neuropathy will lead to better disease management strategies. We aimed to develop a reliable method to re-sequence multiple genes in a large cohort of painful neuropathy patients at low cost. In this study, we compared sensitivity, specificity, targeting efficiency, performance and cost effectiveness of Molecular Inversion Probes-Next generation sequencing (MIPs-NGS) and TruSeq® Custom Amplicon-Next generation sequencing (TSCA-NGS). Capture probes were designed to target nine sodium channel genes (*SCN3A*, *SCN8A-SCN11A*, and *SCN1B-SCN4B)*. One hundred sixty-six patients with diabetic and idiopathic neuropathy were tested by both methods, 70 patients were validated by Sanger sequencing. Sensitivity, specificity and performance of both techniques were comparable, and in agreement with Sanger sequencing. The average targeted regions coverage for MIPs-NGS was 97.3% versus 93.9% for TSCA-NGS. MIPs-NGS has a more versatile assay design and is more flexible than TSCA-NGS. The cost of MIPs-NGS is >5 times cheaper than TSCA-NGS when 500 or more samples are tested. In conclusion, MIPs-NGS is a reliable, flexible, and relatively inexpensive method to detect genetic variations in a large cohort of patients. In our centers, MIPs-NGS is currently implemented as a routine diagnostic tool for screening of sodium channel genes in painful neuropathy patients.

## Introduction

Over the last decade, the field of molecular genetic diagnostic has undergone tremendous changes. Introduction of next generation sequencing (NGS) enabled replacement of single gene tests with comprehensive gene panels, whole exome sequencing (WES) and whole genome sequencing (WGS) to increase the likelihood of identifying causal variants while decreasing the number of tests [1–4].

Currently, there is a growing clinical use of WES and WGS to identify causal variants and to discover new disease related genes in patients. However, routine sequencing of large numbers of WES or WGS remains expensive for clinical use. Several issues such as incidental findings that pose significant ethical problems, cost effectiveness and technical challenges of clinical interpretation of enormous numbers of genetic variants remain challenging [5, 6]. Moreover, WGS generates a huge amount of data that require complex bioinformatic analysis tools for data handling and storage [1].

To fully leverage the power of NGS in a large number of samples in a cost- and time-effective manner, several targeted enrichment approaches are available [7, 8]. Many diagnostic laboratories are implementing targeted enrichment NGS methods to focus on specific genes panels, or genomic regions for genetically heterogeneous diseases [9–12]. The most commonly used custom-enrichment approaches are based on capture by hybridization, PCR-based methods (highly multiplexed PCR) and capture by circularization [7, 13–17].

Neuropathic pain is a common feature of peripheral neuropathy that imposes a significant impact on patients’ quality of life and health care costs. Millions of individuals (7–10% of the general population) worldwide suffer from neuropathic pain [18]. However, not all individuals with peripheral neuropathy develop pain, and it is not possible to predict who is more or less susceptible among those with similar risk exposure [19]. Current inability to identify high-risk individuals hinders development and application of therapies to counteract neuropathic pain and to address targeted prevention strategies. Pathogenic variants in voltage-gated sodium channel (VGSC) genes expressed in the peripheral nociceptive pathway such as *SCN9A*, *SCN10A*, and *SCN11A* have been reported to play a key role in neuropathic pain [20–23].

In order to provide a genetic diagnosis in patients with neuropathic pain, identification of causal variants in genes encoding sodium channel subunits as well as other genes involved in painful neuropathy pathway is needed. Resolving the genetic architecture of painful neuropathy will lead to better disease management strategies, risk stratification, and counselling. Therefore, the aim of this study was to develop, validate and implement a reliable technique to rapidly and accurately re-sequence multiple genes in a large cohort of neuropathic pain patients at low cost. We present here the assessment of Molecular Inversion Probes-Next generation sequencing (MIPs-NGS) and TruSeq^®^ Custom Amplicon-Next generation sequencing (TSCA-NGS, Illumina, Inc., San Diego, CA, USA) methods, using a custom gene panel, to identify genetic variants in patients with neuropathic pain. For both methods, we constructed a targeted enrichment kit to capture the coding and exon-flanking intron sequences of nine sodium channel genes (*SCN3A*, *SCN8A-SCN11A*, and *SCN1B-SCN4B*). We applied the two methods to test 166 different patients and systematically compared the sensitivity, specificity, flexibility, targeting efficiency, reproducibility of performance and cost effectiveness of MIPs-NGS and TSCA-NGS approaches.

## Materials and methods

### Patient samples

In total, 166 samples from patients diagnosed with diabetic and idiopathic neuropathy were tested by MIPs-NGS and TSCA-NGS. For 70 of these patients, exons and exon-flanking intron sequences of *SCN9A*, *SCN10A*, and *SCN11A* genes were analyzed by Sanger sequencing to validate MIPs-NGS and TSCA-NGS methods [23]. Local Medical Ethical Committees of Fondazione IRCCS Istituto Neurologico "Carlo Besta" (Italy), Maastricht University Medical Center (the Netherlands), University of Manchester (United Kingdom) and the Deutsche Diabetes Forschungsgesellschaft EV (Germany) approved this study. Informed consent for genetic testing was given by patients to participate in this study.

Genomic DNA was extracted from peripheral blood by using QIAamp DNA Blood Maxi Kit, Puregene^®^ Blood Core Kit (Qiagen, Hilden, Germany) or NucleoSpin^®8^ Blood Isolation kit (Macherey-Nagel, Düren, Germany). Quality and concentration of the DNA was determined by NanoDrop (Thermo Scientific, Wilmington, USA), and Qubit^®^ 2.0 Fluorometer using the Qubit^®^ dsDNA BR assay kit (Life technologies, Bleiswijk, The Netherlands). Isolated DNA was stored with a unique numeric code in the central DNA bank at Maastricht University Medical Centre and IRCCS Foundation “Carlo Besta” Neurological Institute.

### Targeted enrichment, paired-end sequencing and data processing

Targeted enrichment kits were constructed for this study to capture the coding and exon-flanking intron sequences (±20 base pairs [bp]) of nine VGSC genes; *SCN3A*, *SCN8A-SCN11A* and *SCN1B-SCN4B* (data in S1 Table). The probes were designed for the two methods using their respective informatics pipelines and methods are provided in the subsections below. Probe features and sequencing characteristics are given in Table 2.

#### MIPs-NGS

Two hundred and seventy-six molecular inversion probes (MIPs) were designed to capture 37,467 bp that represent all exons and exon-flanking intron sequences of nine VGSC genes. A modified version of MIPgen tool (http://shendurelab.github.io/MIPGEN/) was used to design MIPs. Data in S1 Fig shows a flowchart of the MIPs design using MIPgen, including required MIPgen criteria.

All probes were fixed in to 77-80-mer in length and each MIP contains two targeting arms; an extension arm ranging in length from 16 to 20 nucleotides (nt) and a ligation arm ranging in length from 20 to 24 nt. These arms were joined by a 30 nt common linker sequence which contains two universal PCR primer sites (complimentary probe arms are available upon request). MIPs were designed to have an overlapping of 20 bps and every base in the targeted region should be covered and captured at least by one probe. All MIPs with high arm copy count (>5x) were excluded. Furthermore, 3’ and 5’untranslated regions (3’ and 5’ UTRs) were not included in the design. Common single nucleotide polymorphisms (SNPs) (>1%) in the extension and ligation arms of the MIPs were excluded whenever possible. In some cases where SNPs were not avoidable, two MIPs were designed to match both wildtype and variant genotype for the same locus (n = 12). Constructed BED file of the targeted regions and MIPs corresponding to these nine candidate genes were uploaded to UCSC genome browser (http://genome.ucsc.edu/) in order to validate each designed MIP.

To reduce costs, the standard gap-fill length of 112 nt between the extension and ligation arm (region of interest) of the MIPs [24] was adapted to 220–230 nt. Each MIP contains a unique molecular identifier (UMI) of 5 nt to remove duplicates introduced by PCR amplification and sequencing. Probes were synthesized by Integrated DNA Technologies (IDT, Iowa, USA) and delivered individually. Subsequently, probes were equimolarly pooled and phosphorylated at 5’ end of the probe (data in S1 File).

**Fig 1:**
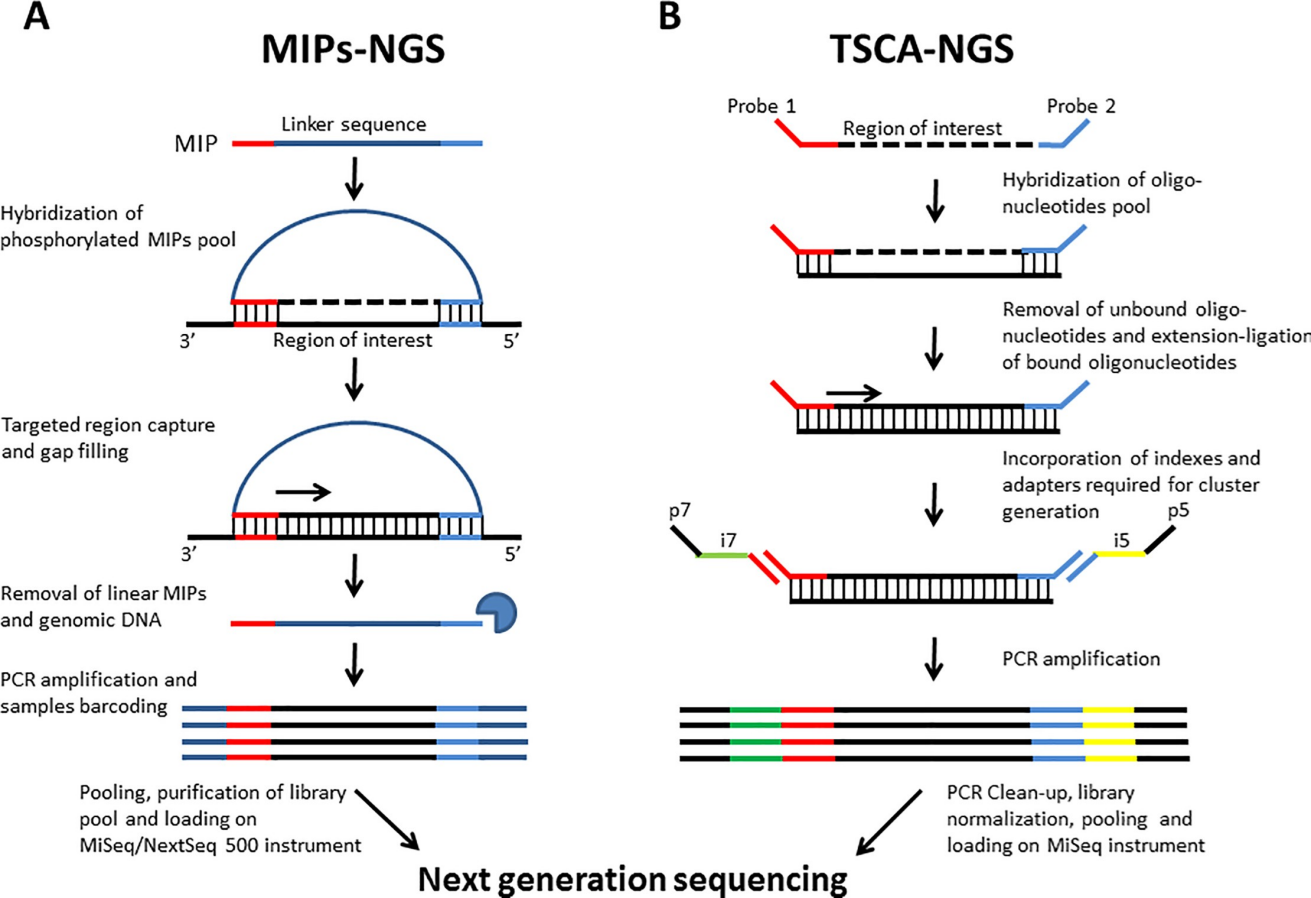
MIPs-NGS and TSCA-NGS workflows summary. Graphical depiction of MIPs-NGS (A) and TSCA-NGS (B) methods for library construction and capture protocol for targeted next-generation sequencing.

Experimental workflow was done by following standard protocols [16, 24, 25, and data in S1 File and S2 Fig]. In brief (Fig 1A), 50–100 ng of high quality, non-fragmented genomic DNA was used for hybridization. After gap filling and ligation, circularized DNA molecules were used as template in PCR with universal primers complementary to the linker sequence. Then, sample-specific barcode sequences and Illumina adaptors were introduced during the PCR amplification step. Fragment size, quality, and quantity of the amplified captured material were determined by Qubit^®^ 2.0 Fluorometer using the Qubit^®^ dsDNA BR assay kit (Life technologies, Bleiswijk, The Netherlands) and Agilent 2100 bioanalyzer using the Agilent DNA 1000 Kit (Agilent Technologies, Santa Clara, CA, USA) following manufacturer’s instructions. Next, samples were pooled and purified using Ampure XP beads (Beckman Coulter, Inc, Brea, California) according to manufacturer’s instructions. Pooled samples were then paired-end sequenced (2 x 150 bp or 2 x 250 bp) using the MiSeq or NextSeq 500 Instrument (Illumina, Inc., San Diego, CA, USA) to achieve >30x coverage per bp.

After a pilot experiment with 10 DNA samples, MIPs with poor performance (15–30 reads) were rebalanced by adding a 5 fold concentration of each poorly performing probe to the original MIPs pool, and MIPs with no sequence reads were replaced by adding new MIPs to the original MIPs pool. Then, the pilot experiment was repeated as described above.

Sequenced data was analyzed by using our in-house MIPs-targeted NGS data analysis pipeline. This pipeline aligns sequenced data to the human reference sequence GRCh37, trims probe arm sequences, de-duplicates the data on the basis of UMIs, and annotates variants according with information and frequencies from ExAC, dbSNP, cadd, Gencode, and calculates coverages per sample, number of bases, mean and median coverage per MIP target [26].

Due to the coverage depth used for this approach, no copy number variant (CNV) analysis has been performed.

#### TSCA-NGS

One hundred and eighty-five oligonucleotides were designed to capture 38,258 bp that represent all exons and exon-flanking intron sequences of nine VGSC genes. The design of oligonucleotides was assessed by the free tool Design Studio, which is available on Illumina website (https://designstudio.illumina.com/ (Illumina Inc., San Diego, CA, USA)). Targeted regions were selected by entering the genes name to the tool and only coding exons were selected. All probes were fixed in to 50-mer in length (including the adapters) and the gap-fill length was set to 425 bp (amplicons size). In case that the tool failed to locate automatically the probes hybridization sites, due to the high CG content or high SNPs rates, we manually added the genomic regions by entering the chromosome coordinates. The *a priori* calculation of the target regions coverage using this tool provided better results when adapting 425 bp amplicon size, instead of the 250 bp option. Increasing the amplicon size resulted in costs reduction in terms of number of oligonucleotides probes spanning the entire target region. One pair of oligonucleotides was designed for each amplicon and provided as a pool in a single tube.

TruSeq Custom Amplicon libraries preparation was performed according to manufacturer’s protocol (TruSeq Custom Amplicon Library Preparation Guide, Part # 15027983 Rev. C). In brief (Fig 1B), 250 ng genomic DNA was hybridized to the custom-made pool of oligonucleotides specific to the targeted regions of interest. Then, unbound oligonucleotides were removed by washing the samples on the provided filtering plate. Subsequently, DNA polymerase was added to extend from the upstream oligonucleotide through the targeted region, followed by ligation to the 5’ end of the downstream oligonucleotide using DNA ligase. The extension-ligation resulted in the formation of products containing the targeted regions of interest flanked by sequences required for PCR amplification. During amplification, the extension-ligation products were amplified using indexed-primers that added sample multiplexing index sequences (i5 and i7) as well as common adapters required for cluster generation (P5 and P7). After purification with PCR Clean-Up AMPure XP beads (Beckman Coulter, Inc.), the library was normalized with Library Normalization Beads. Finally, equal volumes of normalized library were combined, diluted in hybridization buffer, and heat-denatured for sequencing on the MiSeq instrument (paired-end sequenced 2 x 250 bp) (Illumina, Inc., San Diego, CA, USA). No probe dosage adjustment was required for TSCA-NGS.

To identify sequence variations in *SCN3A*, *SCN8A-SCN11A*, *SCN1B-SCN4B* by TSCA-NGS, Raw Fastq files were aligned to reference sequence GRCh37 using CLC Genomics Workbench (CLCbio, Qiagen, Hilden, Germany). Variant calling, annotation and coverage analysis was performed using CLC Genomics Workbench (CLCbio, Qiagen, Hilden, Germany). No CNV analysis was carried out for this approach.

### Sanger sequening

Coding exons and exon-flanking intronic regions of *SCN9A*, *SCN10A* and *SCN11A* of 70 patients were amplified by PCR and sequenced by Sanger sequencing as previously described [23], to assess the sensitivity and specificity both targeted-NGS methods.

### Cross-comparative analysis of genetic variations detection between MIPs-NGS and TSCA-NGS

A cross-comparative analysis was performed in order to compare the sensitivity, specificity and reproducibility of genetic variations detection between MIPs-NGS and TSCA-NGS. We considered only the regions defined in Table in S1 Table as target regions. Regions with high variation rates in exons or mapping at the ends of the reads were excluded to avoid mismatches due to the low quality of reads (Table 1). Variants covered less than 30x, with reads supporting the variant call less than 20%, or that were present in more than 90% of the tested samples were filtered-out to exclude potential artifacts. For the cross-comparative analysis, the overlap between each approach was determined. Disconcordant calls were qualitatively and quantitavely assessed.

**Table 1:** Performance comparison between MIPs-NGS and TSCA-NGS.

Gene name	Targeted region (bp)		Number of probes (n)		Average coverage >30x/bp (%)		Number of probes with no reads (n)	
	MIPs-NGS	TSCA-NGS	MIPs-NGS	TSCA-NGS	MIPs-NGS	TSCA-NGS	MIPs-NGS	TSCA-NGS
SCN3A	7043	7175	55	33	99.6	99.3	0	0
SCN8A	6943	6983	49	33	97.6	97.1	0	2 (ex 12†, 21†)
SCN9A	6934	6974	49	32	98.7	95.8	0	1 (ex 27†)
SCN10A	6871	6951	43	34	99.9	98.6	0	0
SCN11A	6379	6416	46	32	99.9	91.6	1 (ex 1)	1 (ex 1)
SCN1B	967	1216	13	6	93.6	91.3	1 (ex1)	1 (ex1)
SCN2B	768	808	6	5	100.0	93.2	0	0
SCN3B	808	848	6	5	100.0	98.7	0	0
SCN4B	754	887	9	5	86.5	79.7	1 (ex 1)	1 (ex 1)
**Total**	**37467**	**38258**	**276**	**185**	**97.3**	**93.9**	**3**	**6**

†exon partially uncovered.

bp: base pair, ex: exon.

## Results

### Performance of MIPs-NGS and TSCA-NGS

We constructed two targeted enrichment kits, the MIPs-NGS kit which contains 276 probes and TSCA-NGS which has 185 probes to capture all exons and exon-flanking intron sequences (± 20 bp) of the nine VGSC genes *SCN3A*, *SCN8A-SCN11A*, and *SCN1B-SCN4B*. To assess the performance, capture efficiency and sequencing coverage of the on-target regions for these nine VGSC genes, 166 samples were captured and enriched by both methods. Data from this study showed a performance and capture efficiency for the SCN (*SCN3A*, *SCN8A-SCN11A*, and *SCN1B-SCN4B)* MIPs-NGS of 98.9% (n = 273/276 MIPs) compared to 96.7% for the TSCA-NGS (n = 179/185 probes). To increase the overall performance for MIPs-NGS (>30x, number of unique sequencing reads needed to obtain accurate genotype call at certain position), 28 MIPs were rebalanced by adding a 5 fold concentration of each poorly performing probe to the original MIPs pool, and seven MIPs with no sequence reads were replaced by added new MIPs to the original MIPs pool, prior to the testing of the 166 samples. Probes dosage adjustment for TSCA-NGS was unnecessary.

The average targeted regions coverage (coverage >30x/bp) was 97.3% in MIPs-NGS, and 93.9% in TSCA-NGS. Capture efficiencies of individual probes was highly reproducible per region and between different samples for both methods. No sequence reads were obtained for three MIPs (1.1%, n = 3/276) and for six TSCA probes (3.3%, n = 6/185). The first coding exon of *SCN1B*, *SCN4B* and *SCN11A* genes failed completely to be captured and enriched by both approaches. The last coding region (exon 27) of *SCN9A* was partially covered by TSCA-NGS, while it was fully captured by MIP-NGS (Table 1).

Seven exons of *SCN3B* (exon 2), *SCN8A* (exons 13, 16, 21 and 26), *SCN10A* (exon 13) and *SCN11A* (exon 14) tested by MIPs-NGS and three exons of *SCN4B* (exon 2), *SCN10A* (exon 13) and *SCN11A* (exon 14) tested by TSCA-NGS showed high variation in sequencing reads.

### Specificity and sensitivity of MIPs-NGS and TSCA-NGS

To determine the specificity and sensitivity of both targeted NGS approaches, 70 patients were analyzed for sequence variations in *SCN9A*, *SCN10A* and *SCN11A* by Sanger sequencing. Sixty-eight unique variants were identified in these *SCN9A*, *SCN10A* and *SCN11A*, including 65 nucleotide substitutions and three indels, located either in the exonic regions or in the exon-flanking intron sequences (± 20 bp). Sixty-four of the 68 variants are present in both MIPs-NGS and TSCA-NGS sets, including their status being homozygous or heterozygous. Four variants were missed by both methods, as these variants are located within regions with low quality or no coverage (one variant in exon 13 in *SCN10A* and three variants in *SCN11A;* one variant in exon 1 and two variants in exon 14). Based on these results, both capture panels demonstrated a 94.1% sensitivity for variant detection (n = 64/68 variants). When we excluded Sanger sequencing variants located in regions with low quality or no coverage by MIPs-NGS and TSCA-NGS (n = 4 variants), we observed a perfect agreement (100%; no differences in number of variants and zygosity status of variants) between Sanger sequencing data and those obtained using MIPs-NGS and TSCA-NGS (data in S3 Fig; 2 examples for Sanger sequencing validation). By excluding regions with low quality (exons with high variation in reads), the overall false-positive rate in *SCN9A*, *SCN10A* and *SCN11A* was 0% for MIPs-NGS and TSCA-NGS.

### Cross comparative analysis of genetic variations detection

To compare variants calls from MIPs-NGS versus TSCA-NGS, sequence data of the same 166 diabetic and idiopathic neuropathy patients was analyzed with our in-house data analysis pipeline. The overlap in on-target regions from all 166 tested subjects was 3642 (123 unique variants) by MIPs-NGS and 3658 (122 unique variants) by TSCA-NGS. We found that 3642 variant*s* were correctly called by both methods and a true positive call of 99.6% was observed. The 16 dissimilar variants (all the same unique variant present in 16 samples) (0.4%) were due to coverage differences between the two methods (low coverage or the coverage was just below the threshold).

### MIPs-NGS versus TSCA-NGS workflow and costs

MIPs-NGS and TSCA-NGS showed a high multiplexing level and require low sample DNA input (between 50–250 ng). Both protocols are straightforward, but hands-on time and the methodological complexity should be taken into consideration. MIPs-NGS is easier and less time-consuming than TSCA-NGS. The workflow for TSCA-NGS library preparation pass through 86 steps, combining enzymatic and PCR reactions, purification and normalization steps with filer plates and magnetic beads, while MIPs-NGS only required 7 steps to obtain the final library pool [16, 24, 25]. The hands-on time to create a library was 45 minutes for MIPs-NGS versus 130 minutes for TSCA-NGS, regardless of sample size, DNA purity, and concentration of starting material (Table 2 and data in S2 Fig).

**Table 2:** Comparison of recommended DNA input, probe features, sequencing kits, sample processing time for MIPs-NGS and TSCA-NGS.

	MIPs-NGS	TSCA-NGS
Recommended DNA input (ng)	50–100	250
Probe type	DNA; molecular inversion probe	DNA; oligonucleotides
Probe strategy	Multiple amplicons	Multiple amplicons
Length of probes (bp)	77–80 mers	50 mers†
Gap fill length (bp)	220–230	425
Number of probes	276	186
Sequencing kit	2x150/2x250	2x250
Hands on time per library (min)	45	130
Total duration per library (days)	2	1–2

† probes+adapter.

In this study, costs per sample, including probes, chemical reagents and efforts was lower for MIPs-NGS compared to TSCA-NGS. However, when a large number of patients, e.g. 500 samples were included, the costs per sample for MIPs-NGS could drop to > 5 times as cheap as TSCA-NGS (Table 3).

**Table 3:** Price comparison MIPs-NGS and TSCA-NGS on different sequencing platforms.

	MiSeq		NextSeq 500/550	NovaSeq 6000	
Sequencing kit	MiSeq Reagent Kit v2 (300-cycles)	MiSeq Reagent Kit v2 (500-cycles)	NextSeq 500/550 High Output Kit v2.5 (300 cycles)	NovaSeq 6000 SP Reagent Kit (300 cycles)	NovaSeq 6000 SP Reagent Kit (500 cycles)
**MIPs-NGS**					
Sample price† for 100 samples (€)	44.6	45.9	45.5	42.8	46.8
Sample price† for 300 samples (€)	23.5	24.9	24.5	21.7	25.8
Sample price† for 500 samples (€)	19.3	20.7	20.3	17.5	21.6
Sample price† for 1000 samples (€)	16.1	17.5	17.1	14.4	18.4
**TSCA-NGS**					
Sample price‡ for 100 samples (€)	-^1^	163.9	-^1^	-^1^	192.5
Sample price‡ for 300 samples (€)	-^1^	125.0	-^1^	-^1^	-^2^
Sample price‡ for 500 samples (€)	-^1^	113.4	-^1^	-^1^	-^2^
Sample price‡ for 1000 samples (€)	-^1^	103.3	-^1^	-^1^	-^2^

† price per sample based on probes (€5.6/probe), reagents (€1.8), rebalancing, optimization and validation costs (€1575) and sequencing costs (varies from €1073 to €6246). Prices are without VAT, company discounts, labor and equipment costs.

‡ price per sample based on TruSeq® Custom Amplicon Kit v1.5 (€6142-€12488), TruSeq® Custom Amplicon Index Kit (€870) and sequencing costs (€1204-€5162). Prices are without VAT, company discounts, labor and equipment costs.

^1^ not calculated, read length 300-cycles (2 x 150 bp) too short for TSCA-NGS.

^2^ not calculated, maximum number of available sample indexes for TSCA-NGS was n = 96.

## Discussion

Selection of an approach for screening a panel of genes for most laboratories depends on a wide range of criteria, including clinical use of the test, panel size, sensitivity and specificity for the genetic regions of interest, expected number of patients to be tested, turnaround time, approach flexibility and scalability, available equipment, work flow, costs, technical expertise, and availability of bioinformatic support.

In this study, we developed a MIPs-NGS and TSCA-NGS targeted re-sequencing panels for nine sodium channel genes (*SCN3A*, *SCN8A-SCN11A*, and *SCN1B-SCN4B)* known to be associated with neuropathic pain [19, 27], and compared their sensitivity, specificity, targeting efficiency, performance and cost effectiveness. The average targeted regions coverage was 97.3% in MIPs-NGS, and 93.9% in TSCA-NGS. Sensitivity, specificity and performance of both methods were comparable (Table 1). However, MIPs-NGS has a more versatile assay design, and is flexible and cheaper than TSCA-NGS (Tables 2 and 3).

Molecular Inversion Probes (MIPs), also known as padlock probes, belong to the category of molecular techniques that capture sequences by circularization. This technology, first described in 1994, was initially developed for multiplex target discovery and SNP genotyping [28, 29], and has recently been combined with next generation sequencing. TSCA-NGS is an amplicon-based approach for targeted re-sequencing. This approach is based on the design of synthetic oligonucleotides (probes), with complementary sequence to the flanking regions of the target DNA to be sequenced. Amplicon-based sequencing approaches are cheaper than Sanger sequencing, WES and WGS, and characterized by high specificity and deep coverage. Moreover, MIPs-NGS and TSCA-NGS have been successfully employed with good-quality DNA sources such as blood or frozen tissues, and with more challenging samples extracted from formalin-fixed and paraffin-embedded tissues [30].

Development of a sensitive diagnostic custom targeted NGS enrichment kit requires proper design of specific primers or probes for candidate genes. For MIPs-NGS and TSCA-NGS probe design, we used the freely available MIPgen software (http://shendurelab.github.io/MIPGEN/) and Design Studio software of Illumina (https://designstudio.illumina.com/), respectively. Both software tools were user-friendly, and simplify the probe designing process based on the criteria defined by the user.

MIPs-NGS and TSCA-NGS showed high multiplexing level, low DNA input requirements (50-250ng) and no need for DNA shearing compared to other targeted enrichment methods [15, 31].

MIPs-NGS and TSCA-NGS have, in general, straightforward laboratory workflows, however TSCA-NGS pass through many steps compared to MIPs-NGS (data in S2 Fig). The hands-on time per library were 2.9 times shorter for MIPs-NGS compared to TSCA-NGS (Table 2 and data in S2 Fig). In addition, development of automated reaction setups is feasible for MIPs-NGS approach since small number of enzymatic reactions and processing steps are required to achieve targeted region capture and sample barcoding. Such an automated laboratory workflow was recently established for smMIP enrichment to detect genetic variations in *BRCA1* and *BRCA2* [32*]*. Furthermore, this study concluded that smMIPs-NGS has a superior accuracy and turnaround time compared to other genetic testing methods for gene panels or targeted regions [32].

The performance and capture efficiency of MIPs-NGS and TSCA-NGS targeted approaches for *SCN3A*, *SCN8A-SCN11A*, and *SCN1B-SCN4B* was high (98.9% for MIPs-NGS versus 96.7% for TSCA-NGS) and variation per read low; both required for a reliable variant detection. No sequence reads were obtained by MIPs-NGS and TSCA-NGS for the first coding exon of *SCN1B*, *SCN4B* and *SCN11A*. These regions have a high GC content (>50%) which influence the capture efficiency. To avoid false positive variant calling, data of seven MIPs (1678 bp) versus three TSCA probes (794 bp) should be excluded from the analysis. To provide a full coverage for *SCN3A*, *SCN8A-SCN11A*, and *SCN1B-SCN4B*, 2321 bp (6.1%) for MIPs-NGS versus 2467 bp (6.4%) for TSCA-NGS should subsequently be analysed by Sanger sequencing (costs per sample excluding VAT are €29.7 for MIPs-NGS versus €24.75 for TSCA-NGS).

The utility of custom targeted enrichment NGS panels may be limited in some contexts because the kits are inflexible in terms of adding or excluding targeted regions and usually expensive [33]. In many diagnostic laboratories, adjusting existing diagnostic panels have gained popularity specially in investigating genetically heterogeneous disease [34]. MIPs-NGS approach offered a higher degree in flexibility and in optimizing the probe performance compared to TSCA-NGS and other targeted enrichment NGS methods [2, 35]. All MIPs were ordered individually, so each individual MIP can be combined into various panels and can be added to an existing pool. This means that changing the MIP kit content as the regions of interest change over time and to keep up with the ever-increasing numbers of diagnostics requests is possible. Moreover, the performance of poorly performing probes can be improved by individual MIP rebalancing or by redesigning new probes. In our study the performance of MIPs-NGS was improved by adding a 5 fold concentration of each poorly performing probe (n = 28 MIPs) to the existing probe pool without the need to order new probes. TSCA-NGS is less flexible because changing the kit content or optimizing specific probe(s) performance is dependent on manipulations by the supplying company. However, in this study probe dosage adjustment was not needed.

Applying WGS as a comprehensive clinical testing approach is at this time still too expensive. Sequencing the entire human genome is associated with the generation of a massive amount of data and the need of complex downstream data analyses and variants interpretation. The majority of known disease-causing variants are located in exons; thus WES and targeted gene panels are often used to identify clinically relevant variants in known disease genes [36]. WES -nearly- includes all the exonic regions of the human genes, so using WES eliminates the need to select the gene panel content, and exonic variants are more comprehensively assessed. However, the amount of data generated by WES is significantly higher than targeted gene panels. In contrast to WES [36], targeted NGS enrichment gene panels are often used for re-sequencing a selective group of phenotype-specific disease genes in common genetic disorders. This results in more manageable and easier data interpretation, usually with higher quality and depth at lower costs [2].

MIPs-NGS has a higher performance and capture efficiency for *SCN3A*, *SCN8A-SCN11A*, and *SCN1B-SCN4B* compared to the commonly used Agilent SureSelectXT Human All Exon v5 (Illumina) WES enrichment kit (Table in S2 Table). Similar to our data (Tables 1 and 3), several studies have demonstrated that MIPs-NGS is an efficient and inexpensive method for detection of genetic variations in multiple diseases [25, 32, 37, 38]. For example, Zhang *et al*. and Pérez Millán *et al*. presented in their studies the use of MIPs based NGS panels to detect pathogenic mutations in early-onset colorectal cancer patients and in patients with hypopituitarism, respectively [37, 38]. Another potential application of MIPs-NGS is to target noncoding and low covered regions in WES.

High throughput NGS platforms such as NextSeq 500/550 and NovaSeq6000 (Illumina, Inc., San Diego, CA, USA) have enabled reductions in sequencing costs (see Table 3 for comparison costs of sequencing *SCN3A*, *SCN8A-SCN11A*, and *SCN1B-SCN4B* on MiSeq versus NextSeq 500/550 and NovaSeq) and time that have resulted in wide use in diagnostic laboratories. Nevertheless, full capacity high-throughput sequencing runs must be achieved to get the best cost-efficiency (based on maximum number of available sample indexes, 384 samples/run for MIPs-NGS versus 96 samples/run for TSCA-NGS). MIPs-NGS and TSCA-NGS can be easily customized to individual needs. Multiple different targeted MIPs-NGS can be combined cost efficient in one Mid—(48–96 samples) or High output run (96–384 samples) without affecting data quality and coverage for each individual sample by which in diagnostics a turnaround time of 2–4 weeks/sample from arrival to reporting can be achieved.

When designing specific targeted enrichment NGS experiments, total number of samples involved, and cost of probes and reagents must be considered. Our data show that the costs of MIPs-NGS are lower than TSCA-NGS. Using MIPs with a gap-fill length of 220–230 nt [24] and TSCA probes with a region of interest of 425 nt, the cost of MIPs-NGS compared to TSCA-NGS is around 5 times lower per sample (€20.7 for MIPs-NGS versus €113.4 for TSCA-NGS) when 500 samples and more are planned to be tested with MIPs-NGS (Table 3).

In conclusion, our results provide a validation and performance assessment of MIPs-NGS and TSCA-NGS as reliable methods for variant detection in a disease-specific subset of genes. Our results suggests that MIP-NGS is a more flexible and less expensive method for detection of genetic variations and is a reliable screening approach for laboratories involved in diagnostic service for patients with pain-related disorders.

## Supporting information

S1 Fig MIPgen settings and probe features for MIPs design using MIPgen. (A) Flowchart MIPs design, including MIPgen settings and probe features; (B) Schematic presentation of designed MIP; (C) Representative example MIP sequence, extension arm is given in blue, linker including unique molecular identifier (NNNNN) in black, and ligation arm in red. BED, browser extensible data; nt, nucleotide; UTR, untranslated region.
(TIF)

S2 Fig Comparison of hands-on and processing time of MIPs—and TSCA library preparation. MIPs requires for library preparation a hands-on and processing time < 3 working hours, distributed over two days, while TSCA requires > 7 working hours, distributed over 1 day or > 6 working hours, distributed over 2 days. ACD1, Amplicon Control DNA 1; ACP1, Amplicon Control Oligo Pool 1; CAT, Custom Amplicon oligo Tube, containing specific oligos; dNTP, deoxyribonucleotide triphosphate; ELM4, Extension Ligation Mix 4; i5, index i5 adapters; i7, index i7 adapters; LNA1, Library Normalization Additives 1; LNB1, Library Normalization Beads 1; LNS2, Library Normalization Storage Buffer 2; LNW1, Library Normalization Wash 1; OHS2, Oligo Hybridization for Sequencing Reagent 2; p5, p5 primers; p7, p7 primers; PMM2, PCR Master Mix 2; RT, room temperature; SW1, Stringent Wash 1; TDP1, TruSeq DNA Polymerase 1; UB1, Universal Buffer 1.
(TIF)

S3 Fig Detection of sequence variants by MIPs-NGS and Sanger sequencing visualized by Integrative Genomics Viewer (IGV) and Mutation Surveyor (MS). Base mismatches to the Human reference genome hg19 are indicated with an arrow. (A) Homozygous MIPs-NGS variant *SCN9A* c.3448C>T visualized by IGV on reverse complement strand (brown, G) (B) Heterozygous MIPs-NGS variant *SCN10A* c.4984G>A visualized by IGV on reverse complement strand (red, T); (C) Sanger sequencing confirmation of homozygous variant *SCN9A* c.3448C>T visualized by MS; (D) Sanger sequencing confirmation of heterozygous variant *SCN10A* c.4984G>A visualized by MS.
(TIF)

S1 File MIPS-NGS protocol. (PDF)

S1 Table Target regions of MIPs-NGS and TSCA-NGS enrichment panels (human reference sequence GRCh37). (PDF)

S2 Table Comparison of targeted region coverage of MIPs-NGS, TSCA-NGS and WES. (PDF)
