# Correction: Evaluation of molecular inversion probe versus TruSeq® custom methods for targeted next-generation sequencing

**DOI:** 10.1371/journal.pone.0248250

**Published:** 2021-03-02

**Authors:** Rowida Almomani, Margherita Marchi, Maurice Sopacua, Patrick Lindsey, Erika Salvi, Bart de Koning, Silvia Santoro, Stefania Magri, Hubert J. M. Smeets, Filippo Martinelli Boneschi, Rayaz A. Malik, Dan Ziegler, Janneke G. J. Hoeijmakers, Gidon Bönhof, Sulayman Dib-Hajj, Stephen G. Waxman, Ingemar S. J. Merkies, Giuseppe Lauria, Catharina G. Faber, Monique M. Gerrits, on behalf on the PROPANE Study Group

The eleventh author’s middle initial is incorrect. The correct name is: Rayaz A. Malik.
