# Supplementary figures and images for: Evaluation of molecular inversion probe versus TruSeq® custom methods for targeted next-generation sequencing
Source: PLoS One. 2020 Sep 2;15(9):e0238467. doi: 10.1371/journal.pone.0238467 (PMC7467307; doi:10.1371/journal.pone.0238467)

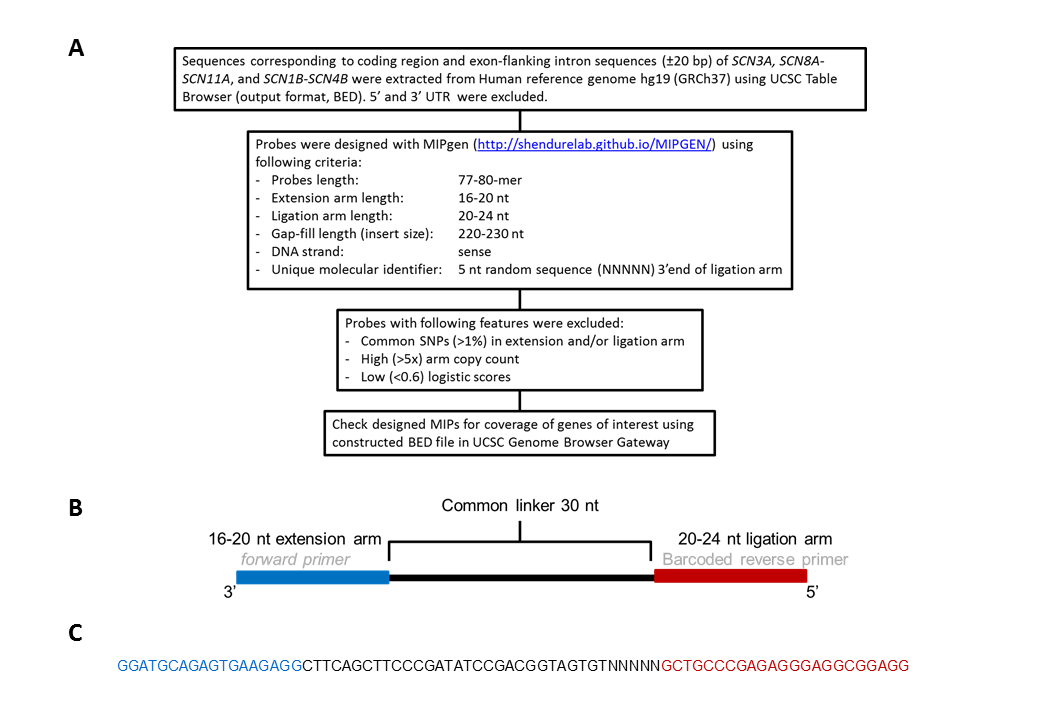

Supplement: S1 Fig — (A) Flowchart MIPs design, including MIPgen settings and probe features; (B) Schematic presentation of designed MIP; (C) Representative example MIP sequence, extension arm is given in blue, linker including unique molecular identifier (NNNNN) in black, and ligation arm in red. BED, browser extensible data; nt, nucleotide; UTR, untranslated region. (TIF) [file pone.0238467.s001.tif]

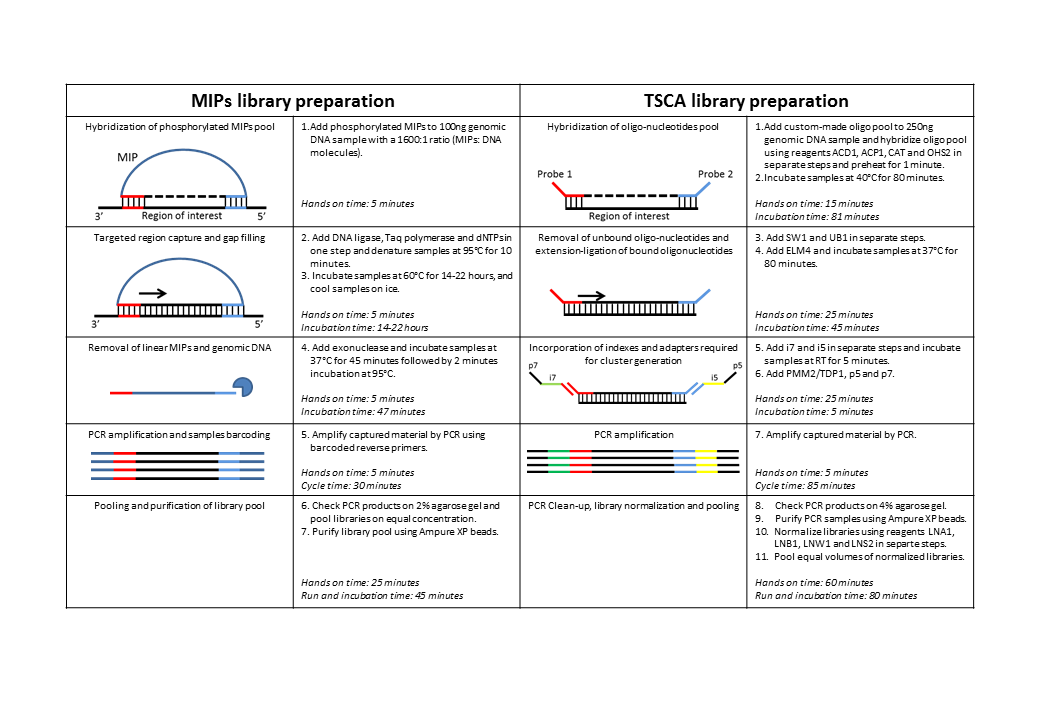

Supplement: S2 Fig — MIPs requires for library preparation a hands-on and processing time < 3 working hours, distributed over two days, while TSCA requires > 7 working hours, distributed over 1 day or > 6 working hours, distributed over 2 days. ACD1, Amplicon Control DNA 1; ACP1, Amplicon Control Oligo Pool 1; CAT, Custom Amplicon oligo Tube, containing specific oligos; dNTP, deoxyribonucleotide triphosphate; ELM4, Extension Ligation Mix 4; i5, index i5 adapters; i7, index i7 adapters; LNA1, Library Normalization Additives 1; LNB1, Library Normalization Beads 1; LNS2, Library Normalization Storage Buffer 2; LNW1, Library Normalization Wash 1; OHS2, Oligo Hybridization for Sequencing Reagent 2; p5, p5 primers; p7, p7 primers; PMM2, PCR Master Mix 2; RT, room temperature; SW1, Stringent Wash 1; TDP1, TruSeq DNA Polymerase 1; UB1, Universal Buffer 1. (TIF) [file pone.0238467.s002.tif]

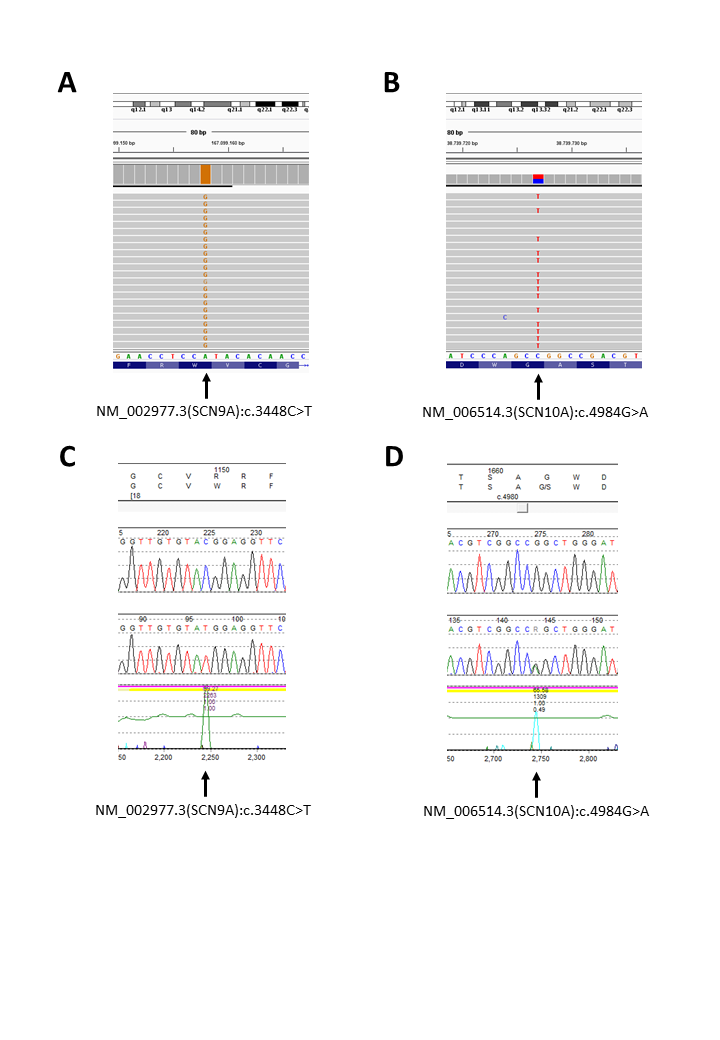

Supplement: S3 Fig — Base mismatches to the Human reference genome hg19 are indicated with an arrow. (A) Homozygous MIPs-NGS variant SCN9A c.3448C>T visualized by IGV on reverse complement strand (brown, G) (B) Heterozygous MIPs-NGS variant SCN10A c.4984G>A visualized by IGV on reverse complement strand (red, T); (C) Sanger sequencing confirmation of homozygous variant SCN9A c.3448C>T visualized by MS; (D) Sanger sequencing confirmation of heterozygous variant SCN10A c.4984G>A visualized by MS. (TIF) [file pone.0238467.s003.tif]
