# Supplementary material for: Evaluation of molecular inversion probe versus TruSeq® custom methods for targeted next-generation sequencing
Source: PLoS One. 2020 Sep 2;15(9):e0238467. doi: 10.1371/journal.pone.0238467 (PMC7467307; doi:10.1371/journal.pone.0238467)
Supplement: S1 File — (PDF) [file pone.0238467.s004.pdf]

MIPs-NGS protocol

MIPgen (http://shendurelab.github.io/MIPGEN/) was used to design MIPs to capture 37,467 bp that represent the coding region and exon-flanking intron sequences (±20) of *SCN3A*, *SCN8A-SCN11A*, *SCN1B-SCN4B*. Probes were synthesized by IDT (Integrated DNA Technologies; Coralville, USA). Probes were equimolarly pooled and phosphorylated at 5'end of the probe using 10U T4 Polynucleotide Kinase (NEB, USA) per 25 µl of 100 µM of MIPs. Based on average coverage and performance of all individual probes, a rebalanced MIPs pool was prepared by adding a 5 fold concentration of each poorly performing probe and a new probe for each probe with no sequence reads, in order to increase coverage uniformity and performance efficiency of all MIPs.

Phosphorylated MIPs were added to 100ng genomic DNA with a 1600:1 ratio (MIPs:DNA molecules) to capture target regions of interest in a mixture of 25 µl, containing 1X Ampligase reaction Buffer (Epicentre, USA), 0.025mM dNTPs (GE Healthcare), 1U Ampligase® DNA Ligase (Epicentre, USA), 3.2U Hemo Klentaq® (NEB, USA) and H_2O. Then, the samples were denatured at 95°C for 10 minutes and incubated at 60°C for 14-22 hours. After incubation, samples were cooled on ice to stop the reaction, and subsequently treated by exonuclease to remove non-circular molecules (linear probes and the remaining single-stranded DNA). Exonuclease treatment was performed by adding for each sample 10U Exonuclease I (NEB, USA), 50 U exonuclease III (NEB) and 1X Ampligase reaction Buffer (Epicentre, USA) and H_2O. Then, the samples were incubated at 37°C for 45 minutes, followed by 2 minutes incubation at 95°C.

Subsequently, captured material was amplified by polymerase chain reaction (PCR) using barcoded reverse primers [1]. In brief, PCR was performed in a final volume of 25 µl in an iCycler Thermal Cycler (Bio-Rad, Hercules, CA, USA). Each reaction contained 5 µl of exonuclease treated MIPs sample, 1.25 µl of barcoded reverse primer (10 µM) [1], 0.125 µl of forward primer (100 µM) [1], 6.125 µl of H_2O and ready to use 2X iPROOF HF master mix

solution (Bio-Rad, Hercules, CA, USA). Reaction mixtures were initially denatured at 98°C for 30 seconds, followed by 22 cycles of 98°C for 10 seconds (total number of PCR cycles was determined by Real time PCR), 60°C for 30 seconds, and 72°C for 30 seconds, and a final extension step at 72°C for 2 minutes.

After PCR amplification, PCR products were tested on 2% agarose gel and then pooled prior for purification by using Ampure XP beads (Beckman Coulter, Inc, Brea, California) according to manufacturer's instructions. Resulting libraries were sequenced using an Illumina MiSeq or NextSeq 500 system, with 2 × 150-bp or 2 × 250-bp paired-end reads.

Reference

1. Arts P, van der Raadt J, van Gestel SHC, Steehouwer M, Shendure J, Hoischen A, Albers CA. Quantification of differential gene expression by multiplexed targeted resequencing of cDNA. Nat commun. 2017;8:15190. dio: 10.1038/ncomms15190
