# Supplementary material for: Evaluation of molecular inversion probe versus TruSeq® custom methods for targeted next-generation sequencing
Source: PLoS One. 2020 Sep 2;15(9):e0238467. doi: 10.1371/journal.pone.0238467 (PMC7467307; doi:10.1371/journal.pone.0238467)
Supplement: S1 Table — (PDF) [file pone.0238467.s005.pdf]

S1 Table: Target regions of MIPs-NGS and TSCA-NGS enrichment panels (human reference sequence GRCh37).

Gene id	Exon number†	Chromosome	Genomic start position target region ‡	Genomic end position target region ‡	Excluded
SCN3A	1	2	-	-	5'UTR
SCN3A	2	2	-	-	5'UTR
SCN3A	3	2	166032924	166032621	5'UTR part
SCN3A	4	2	166027078	166026920	
SCN3A	5	2	166025355	166025226	
SCN3A	6	2	166021050	166020882	
SCN3A	7	2	166020239	166020108	
SCN3A	7	2	166020423	166020292	
SCN3A	8	2	166019358	166019046	
SCN3A	9	2	166018901	166018798	
SCN3A	10	2	166012433	166012252	
SCN3A	11	2	166011188	166010942	
SCN3A	12	2	166003559	166003229	
SCN3A	13	2	165997528	165997141	
SCN3A	14	2	165996138	165995966	
SCN3A	15	2	165994647	165994369	
SCN3A	16	2	165987947	165987734	
SCN3A	18	2	165984631	165984121	
SCN3A	19	2	165972105	165971945	
SCN3A	20	2	165970500	165970306	
SCN3A	21	2	165969588	165969375	
SCN3A	22	2	165956954	165956792	
SCN3A	23	2	165954054	165953742	
SCN3A	24	2	165953050	165952957	
SCN3A	25	2	165952178	165952001	
SCN3A	26	2	165951008	165950864	
SCN3A	27	2	165949054	165948744	
SCN3A	28	2	165947875	165946640	3'UTR part
SCN8A	1	12	-	-	5'UTR
SCN8A	2	12	52056582	52056897	
SCN8A	3	12	52077938	52078096	
SCN8A	4	12	52080132	52080261	
SCN8A	5	12	52080855	52081023	
SCN8A	6	12	52082522	52082653	
SCN8A	7	12	52093334	52093595	

SCN8A	8	12	52094908	52095011	
SCN8A	9	12	52096537	52096718	
SCN8A	10	12	52099181	52099427	
SCN8A	11	12	52100186	52100519	
SCN8A	12	12	52115310	52115712	
SCN8A	13	12	52139667	52139839	
SCN8A	14	12	52145119	52145397	
SCN8A	15	12	52156267	52156480	
SCN8A	16	12	52159435	52159831	
SCN8A	17	12	52162629	52163139	
SCN8A	18	12	52163632	52163789	
SCN8A	19	12	52164293	52164487	
SCN8A	20	12	52167953	52168166	
SCN8A	21	12	52174414	52174587	
SCN8A	22	12	52180306	52180630	
SCN8A	23	12	52182459	52182552	
SCN8A	24	12	52183045	52183222	
SCN8A	25	12	52184162	52184306	
SCN8A	26	12	52188135	52188445	
SCN8A	27	12	52200046	52201232	3’UTR part
SCN9A	1	2	-	-	5’UTR
SCN9A	2	2	167168286	167167989	
SCN9A	3	2	167163604	167163446	
SCN9A	4	2	167163129	167163000	
SCN9A	5	2	167162450	167162282	
SCN9A	6	2	167160859	167160728	
SCN9A	7	2	167159832	167159580	
SCN9A	8	2	167151192	167151089	
SCN9A	9	2	167149902	167149721	
SCN9A	10	2	167145173	167144927	
SCN9A	11	2	167143153	167142826	
SCN9A	12	2	167141354	167140976	
SCN9A	13	2	167138338	167138169	
SCN9A	14	2	167137125	167136847	
SCN9A	15	2	167134843	167134630	
SCN9A	16	2	167133869	167133473	
SCN9A	17	2	167129405	167128889	
SCN9A	18	2	167108415	167108255	
SCN9A	19	2	167099186	167098992	

SCN9A	20	2	167094797	167094584	
SCN9A	21	2	167089992	167089830	
SCN9A	22	2	167085502	167085181	
SCN9A	23	2	167084253	167084160	
SCN9A	24	2	167083234	167083057	
SCN9A	25	2	167060994	167060850	
SCN9A	26	2	167060755	167060445	
SCN9A	27	2	167056394	167055162	3’UTR part
SCN10A	1	3	38835521	38835212	
SCN10A	2	3	38833679	38833521	
SCN10A	3	3	38830547	38830427	
SCN10A	4	3	38812918	38812750	
SCN10A	5	3	38805107	38804976	
SCN10A	6	3	38802894	38802663	
SCN10A	7	3	38802258	38802152	
SCN10A	8	3	38798670	38798489	
SCN10A	9	3	38798382	38798145	
SCN10A	10	3	38797469	38797259	
SCN10A	11	3	38794023	38793690	
SCN10A	12	3	38791695	38791544	
SCN10A	13	3	38784040	38783762	
SCN10A	14	3	38781199	38780986	
SCN10A	15	3	38770412	38770013	
SCN10A	16	3	38768563	38768077	
SCN10A	17	3	38766825	38766645	
SCN10A	18	3	38765064	38764901	
SCN10A	19	3	38763923	38763729	
SCN10A	20	3	38760337	38760124	
SCN10A	21	3	38755591	38755429	
SCN10A	22	3	38753956	38753632	
SCN10A	23	3	38752408	38752315	
SCN10A	24	3	38751126	38750949	
SCN10A	25	3	38748894	38748750	
SCN10A	26	3	38743620	38743310	
SCN10A	27	3	38740073	38738820	
SCN11A	1	3	38991873	38991567	5’UTR part
SCN11A	2	3	38988418	38988258	
SCN11A	3	3	38987021	38986882	
SCN11A	4	3	38968442	38968274	

SCN11A	5	3	38967020	38966886	
SCN11A	6	3	38962766	38962547	
SCN11A	7	3	38961512	38961406	
SCN11A	8	3	38951718	38951537	
SCN11A	9	3	38950705	38950468	
SCN11A	10	3	38949633	38949420	
SCN11A	11	3	38946832	38946663	
SCN11A	12	3	38945614	38945336	
SCN11A	13	3	38941584	38941365	
SCN11A	14	3	38938736	38938316	
SCN11A	15	3	38936475	38936004	
SCN11A	16	3	38927749	38927596	
SCN11A	17	3	38926913	38926759	
SCN11A	18	3	38924898	38924704	
SCN11A	19	3	38921634	38921421	
SCN11A	20	3	38913805	38913664	
SCN11A	21	3	38913219	38912916	
SCN11A	22	3	38912255	38912162	
SCN11A	23	3	38908969	38908792	
SCN11A	24	3	38904810	38904666	
SCN11A	25	3	38892262	38891952	
SCN11A	26	3	38889253	38888165	
SCN1B	1	19	35521705	35521784	5’UTR part
SCN1B	2	19	35523412	35523606	
SCN1B	3	19	35524383	35525010	
SCN1B	3A*	19	35524383	35525022	3’UTR part
SCN1B	4	19	35530001	35530170	
SCN1B	5	19	35530519	35530613	
SCN2B	1	11	118047166	118047057	5’UTR part
SCN2B	2	11	118039486	118039280	
SCN2B	3	11	118039030	118038780	
SCN2B	4	11	118037821	118037582	3’UTR part
SCN3B	1	11	123524529	123524435	5’UTR part
SCN3B	2	11	123516478	123516275	
SCN3B	3	11	123513399	123513134	
SCN3B	4	11	123509052	123508874	
SCN3B	5	11	123504934	123504831	3’UTR
SCN4B	1	11	118023408	118023308	5’UTR part
SCN4B	2	11	118015964	118015752	

SCN4B	3	11	118014796	118014528	
SCN4B	4	11	118012071	118011902	
SCN4B	5	11	118007855	118007722	3'UTR part

† Exons numeration is referred to the selected transcript: *SCN3A* (NM_006922.3); *SCN8A* (NM_014191.3), *SCN9A* (NM_002977.3), *SCN10*A (NM_006514.3), *SCN11A* (NM_014139.2), *SCN1B* (NM_001037.4 and * isoform NM_199037.4), *SCN2B* (NM_004588.4), *SCN3B* (NM_018400.3), *SCN4B* (NM_174934.3).
‡ Target regions include only coding and exon-flanking intron sequences ±20 bp. UTRs were excluded for both approaches.
Id, identifier; UTR, untranslated region.
