# Supplementary material for: Evaluation of molecular inversion probe versus TruSeq® custom methods for targeted next-generation sequencing
Source: PLoS One. 2020 Sep 2;15(9):e0238467. doi: 10.1371/journal.pone.0238467 (PMC7467307; doi:10.1371/journal.pone.0238467)
Supplement: S2 Table — (PDF) [file pone.0238467.s006.pdf]

S2 Table. Comparison of targeted region coverage of MIPs-NGS, TSCA-NGS and WES.

Gene name	Targeted region coverage†		
	MIPs-NGS (%)	TSCA-NGS (%)	WES (%)‡
SCN3A	99.6	99.3	95.5
SCN8A	97.6	97.1	98.3
SCN9A	98.7	95.8	94.9
SCN10A	99.9	98.6	98.8
SCN11A	99.9	91.6	95.0
SCN1B	93.6	91.3	94.7
SCN2B	100.0	93.2	100
SCN3B	100.0	98.7	100
SCN4B	86.5	79.7	89.1
**Total**	**97.3**	**93.9**	**96.5**

† Sufficient targeted region coverage is defined as >30x/bp for MIPs-NGS and TSCA-NGS, and >20x/bp for WES (general standard cut-off values used for these approaches).
‡ Results are based on HiSeq exome sequencing data of 100 exomes enriched with Agilent SureSelectXT Human All Exon v5 kit (Illumina).
